# p38α MAPK integrates opposing oncogenic APC/β-catenin/TCF and GUCY2C tumor-suppressor axes in colorectal cancer

**DOI:** 10.64898/2026.10.05.756833

**Published:** 2026-10-06

**Authors:** Ariana A. Entezari, Adi Caspi, Jasmine R. Alvarez, Andrew E. Evans, Jeffrey A. Rappaport, Thomas J.M. Kuret, Allison S. Doermann, Ramkrishna Mitra, Adam E. Snook, Scott A. Waldman

## Abstract

**Introduction:**

Sporadic colorectal cancers (CRCs) form following initiating APC mutations, inducing constitutive β-catenin/TCF signaling. Similarly, the guanylyl cyclase C (GUCY2C) tumor suppressor axis is universally silenced in CRC. While the GUCY2C receptor is retained, loss of its paracrine hormones guanylin (GUCA2A) and uroguanylin (GUCA2B) is among the earliest events in transformation. Mutant APC/β-catenin/TCF signaling driving CRC transcriptionally silences GUCY2C hormone expression while suppressing that oncogenic pathway reconstitutes hormone production opposing tumorigenesis. p38α opposes tumor initiation, and its loss promotes transformation in chemical and genetic mouse models of CRC. In healthy intestine, p38α supports a gradient of epithelial differentiation along the crypt-surface axis. Here, we reveal that p38α links regulation of oncogenic APC/β-catenin/TCF signaling and GUCY2C hormone expression.

**Methods:**

Regulation of p38α by APC/β-catenin/TCF signaling was defined in four genetically engineered human CRC cell lines with conditional oncogenic APC/β-catenin/TCF signaling and healthy human colon organoids. Expression of p38α was eliminated from conditional human CRC cell lines by pharmacological inhibition, RNA knockdown, and CRISPR/Cas9 gene editing. Transcriptomic analysis of these cells by RNA sequencing defined the set of genes regulated by APC/β-catenin/TCF signaling controlled through p38α.

**Results:**

In CRC cell lines, APC/β-catenin/TCF signaling suppressed p38α phosphorylation and activity, whereas inhibition of APC/β-catenin/TCF restored p38α signaling. In the absence of APC/β-catenin/TCF signaling, inhibiting p38α repressed GUCY2C hormone expression. Inhibiting APC/β-catenin/TCF signaling enriched phosphorylated p38α at the GUCA2A promoter. In that context, analysis of the CPTAC proteomic database revealed that phosphorylation of p38α and its upstream kinases MKK3 and MKK4 was reduced in human CRC. Finally, transcriptomic analysis revealed that p38α is a critical signaling node controlling the expression of a substantial subset of APC/β-catenin/TCF-regulated genes in human CRC cells and patient tumors.

**Conclusions:**

APC/β-catenin/TCF signaling inhibits p38α phosphorylation and transcriptional activity, suppressing GUCA2A and GUCA2B expression and functionally silencing the retained GUCY2C receptor. These findings identify p38α as a signaling intermediary integrating oncogenic APC/β-catenin/TCF activity to extinction of the GUCY2C tumor-suppressor axis and reveal a broader p38α-dependent component of the APC/β-catenin/TCF-regulated transcriptome.

**Abstract Figure:**

## Full Text Availability

The license terms selected by the author(s) for this preprint version do not permit archiving in PMC. The full text is available from the preprint server.

